# Significant Correlation Between Grip Strength and m2bpgi in Patients with Chronic Liver Diseases

**DOI:** 10.3390/jcm8091359

**Published:** 2019-09-01

**Authors:** Hiroki Nishikawa, Hirayuki Enomoto, Kazunori Yoh, Yoshinori Iwata, Yoshiyuki Sakai, Kyohei Kishino, Naoto Ikeda, Tomoyuki Takashima, Nobuhiro Aizawa, Ryo Takata, Kunihiro Hasegawa, Noriko Ishii, Yukihisa Yuri, Takashi Nishimura, Hiroko Iijima, Shuhei Nishiguchi

**Affiliations:** 1 Division of Hepatobiliary and Pancreatic disease, Department of Internal Medicine, Hyogo College of Medicine, Nishinomiya, Hyogo 663-8501, Japan; 2 Center for Clinical Research and Education, Hyogo College of Medicine, Nishinomiya, Hyogo 663-8501, Japan

**Keywords:** Grip strength, Chronic liver disease, M2BPGi, FIB4 index, Correlation

## Abstract

We sought to compare the impact upon grip strength (GS) between the Mac-2 binding protein glycosylation isomer (M2BPGi) and the Fibrosis-4 (FIB4) index in chronic liver disease (CLD) patients (*n* = 376: 171 males and 205 females, and 137 liver cirrhosis (LC) cases (36.4%)). Factors linked to the low GS (<26 kg in male and <18 kg in female) were also investigated using univariate and multivariate analyses. The median GS in males was 35.5 kg, while that in females was 21.1 kg. The median M2BPGi was 1.11 cutoff index, whereas the median FIB4 index was 2.069. In both male (*P* < 0.0001) and female (*P* = 0.0001), GS in LC patients was significantly lower than that in non-LC patients. In males, M2BPGi (*r* = −0.4611, *P* < 0.0001) and the FIB4 index (*r* = −0.4556, *P* < 0.0001) significantly correlated with GS. Similarly, in females, M2BPGi (*r* = −0.33326, *P* < 0.0001) and our FIB4 index (*r* = −0.26388, *P* = 0.0001) also significantly correlated with GS. In the multivariate analyses of factors linked to the low GS, independent factors were: M2BPGi (*P* = 0.0003) and skeletal muscle index (*P* = 0.0007) in males, and age (*P* < 0.0001) and serum albumin level (*P* = 0.0484) in females. In conclusion, liver fibrosis markers were well-correlated with GS in CLD patients. In particular, M2BPGi can be helpful for predicting the low GS in male patients.

## 1. Introduction

Grip strength (GS) is a parameter of muscle function, and muscle dysfunction, such as decreased GS, has been suggested to be a key mechanism of fatigue in chronic liver diseases (CLDs) [1,2]. GS is also an easy and reproducible indication with respect to frailty measures [3]. The clinical implication of GS in patients with CLDs has been verified in several studies. In our previous investigation, we reported that GS had a strong influence especially in the physiological domains in the 36-Item Short-Form Health Survey in patients with CLDs [4]. We also demonstrated that muscle strength decline was closely associated with sleep disorder in patients with CLDs [5], while non-alcoholic fatty liver disease (NAFLD) is reported to be linked to muscular impairment in elderly adults [6,7,8]. Lee et al. reported that there were linear decreases in the NAFLD index across incremental GS values [8]. A previous large, multicenter, longitudinal population study with varying incomes and sociocultural backgrounds, showed that the measurement of GS is a simple, inexpensive risk-stratifying screening tool for all-cause death, cardiovascular-related death, and cardiovascular disease [9]. Thus, factors relevant to decreased GS are of importance in the clinical settings.

A novel liver fibrosis marker (Mac-2 binding protein glycosylation isomer (M2BPGi)), which is a glycobiomarker associated with CLD-related liver fibrosis with a unique fibrosis-related glycoalteration, has been recently established by Japanese researchers [10,11,12,13]. The usefulness of M2BPGi for the prediction of the severity of liver fibrosis or liver carcinogenesis has been well confirmed, while the FIB4 index is also a well-validated liver fibrosis marker in patients with CLDs [13,14,15,16,17,18,19,20,21]. A recent large observational study reported that patients with liver cirrhosis (LC) before a sustained virological response (SVR) to treatment for the hepatitis C virus (HCV) infection continue to have a significant risk for hepatocellular carcinoma (HCC) development (over 2% per year) for a large number of years, even when their FIB4 index decreases, and should continue HCC surveillance, while non-LC patients with FIB4 index 3.25 or more have a higher risk to merit HCC surveillance, especially when FIB4 index remains 3.25 or more post-SVR [19]. 

However, there have been no reports examining the impact of M2BPGi or FIB4 index on GS in CLD patients. In this study, we sought to compare the impact on GS between M2BPGi and FIB4 index in CLD patients. 

## 2. Patients and Methods

### 2.1. Patients

A total of 376 chronic liver disease (CLD) individuals with data for both grip strength (GS) and Mac-2 binding protein glycosylation isomer (M2BPGi) were admitted to our hospital between February 2014 and January 2019, and they were subjected to this analysis. GS was tested based upon the current Japanese guidelines [22]. For the measurement of GS, we used a Smedley grip dynamometer. The dynamometer was placed so that the needle was on the lateral side, and the patient stood upright with both of their legs spread naturally, and both arms hanging down naturally. The measurement was done in this state by having the patient firmly grip the device while making sure it did not come into contact with the body or with the clothing. Two measurements were done on both sides. The better measurement on each side was chosen, and GS was calculated as the average of these values [22]. Liver cirrhosis (LC) was determined based on pathological data, radiologic findings and/or laboratory data [23,24,25,26]. HCC diagnosis was based on previous reports [27]. M2BPGi was measured as reported elsewhere [28]. The fibrosis-4 (FIB4) index was calculated as reported previously [29]. The skeletal muscle index (SMI, kg/m^2^) was measured using bioimpedance analysis (BIA), as reported elsewhere [30]. Patients with severe ascites were excluded because of the lack of reliability for BIA testing. We retrospectively examined the relationship between the baseline GS value and clinical parameters. The low GS was defined as <26 kg in male and <18 kg in female based on the current guidelines [22,31]. Factors linked to the low GS were also investigated using univariate and multivariate analyses. 

This study protocol was acknowledged by the institutional review board in the Hyogo College of Medicine Hospital (approval no. 1831) and the Declaration of Helsinki was strictly followed in order to guarantee the right of the study subjects. All patients provided written informed consent. Personal information was protected during data collection. 

### 2.2. Statistical Considerations

In the analysis of continuous parameters, we employed Student’s *t* test, the Mann-Whitney U test, Pearson’s correlation coefficient *r*, analysis of variance or Kruskal-Wallis test to assess group difference, as appropriate. In the analysis of categorical parameters, we employed a Pearson χ^2^ test to assess group difference. Baseline items significantly correlated with the low GS in our univariate analysis were also entered into the multivariate logistic regression analysis to select candidate items. Unless otherwise stated, data were presented as a median value (interquartile range (IQR)). We set the threshold for statistical significance at *P* < 0.05. The JMP 14 (SAS Institute Inc., Cary, NC, USA) was employed to analyze statistically.

## 3. Results

### 3.1. Baseline Characteristics

The demographic and clinical characteristics of the analyzed subjects (*n* = 376) were demonstrated in Table 1. The study cohort included 171 males and 205 females with the median age (IQR) of 64 (52, 71) years. In the etiologies for CLD, HCV was in the majority (208/376, 55.3%). The median (IQR) GS in male was 35.5 kg (29.2 kg, 41.5 kg), while that in female was 21.1 kg (17.9 kg, 24.4 kg). The median (IQR) M2BPGi was 1.11 cutoff index (COI) (0.70 COI, 2.34 COI), whereas the median (IQR) FIB4 index was 2.069 (1.2515, 3.451). LC and HCC were identified at baseline in 137 cases (36.4%) and 9 cases (2.4%), respectively. The median (IQR) GS (36.6 kg (30.725 kg, 41.975 kg)) in male Child-Pugh A patients (*n* = 152) was significantly higher than that (25.7 kg (21.4 kg, 29.9 kg)) in male Child-Pugh B or C patients (*n* = 19) (*P* < 0.0001). The median (IQR) GS (21.4 kg (18.25 kg, 24.5 kg)) in female Child-Pugh A patients (*n* = 193) was significantly higher than that (18.1 kg (15.475 kg, 19.1 kg)) in female Child-Pugh B or C patients (*n* = 12) (*P* = 0.0034). 

### 3.2. Gs According to the LC Status in Male and Female

**Figure 1:**
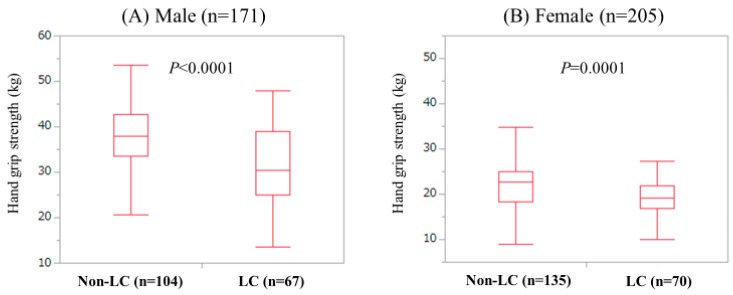
Grip strength (GS) value according to the liver cirrhosis (LC) status in males (**A**) and females (**B**).

In the male, GS in LC patients (median (IQR) = 30.5 kg (25.1 kg, 39.0 kg)) was significantly lower than that in non-LC patients (median (IQR) = 37.85 kg (33.575 kg, 42.775 kg)). (*P* < 0.0001) (Figure 1A) The proportion of LC patients with low GS (28.36%, 19/67) was significantly higher than that of non-LC patients with low GS (5.77%, 6/104) (*P* < 0.0001). In the female, GS in LC patients (median (IQR) = 19.1 kg (16.775 kg, 21.8 kg)) was significantly lower than that in non-LC patients (median (IQR) = 22.7 kg (18.3 kg, 25.0 kg)) (*P* = 0.0001). (Figure 1B) The proportion of LC patients with low GS (30.0%, 21/70) was not significantly higher than that of non-LC patients with low GS (22.96%, 31/135) (*P* = 0.3109).

### 3.3. GS According to Age in Male and Female

**Figure 2:**
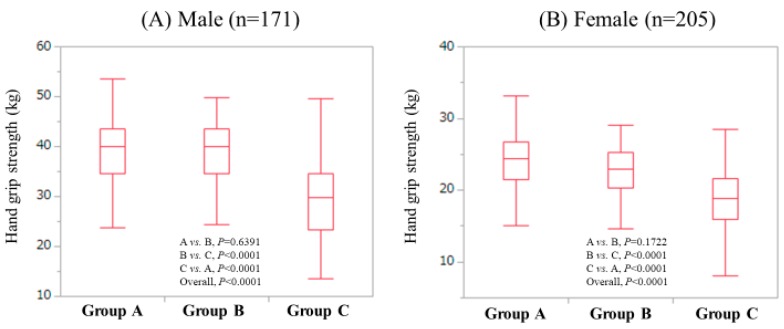
GS value according to the LC status in male (**A**) and female (**B**); Group A indicates patients 50 or less than 50 years; Group B indicates patients more than 50 years and 65 or less than 65 years; Group C indicates patients more than 65 years.

Patients were classified into three groups according to age: <50 years (group A); ≥50 years and <65 years (group B); and ≥65 years (group C). In males, the median (IQR) GS in groups of A (*n* = 72), B (*n* = 46) and C (*n* = 53) were: 39.9 kg (34.6 kg, 43.6 kg), 40.05 kg (34.65 kg, 43.6 kg) and 29.75 kg (23.425 kg, 34.55 kg), respectively. The overall difference was noted with significance (*P* < 0.0001: A vs. B, *P* = 0.6391; B vs. C, *P* < 0.0001; A vs. C, *P* < 0.0001). (Figure 2A) In females, the median (IQR) GS in groups of A (*n* = 31), B (*n* = 67) and C (*n* = 101) were: 24.4 kg (21.5 kg, 26.7 kg), 23.0 kg (20.3 kg, 25.2 kg) and 18.9 kg (16.0 kg, 21.6 kg), respectively. An overall difference was identified with significance (*P* < 0.0001: A vs. B, *P* = 0.1722; B vs. C, *P* < 0.0001; A vs. C, *P* < 0.0001). (Figure 2B)

### 3.4. GS According to Body Mass Index (BMI) in Male and Female

**Figure 3:**
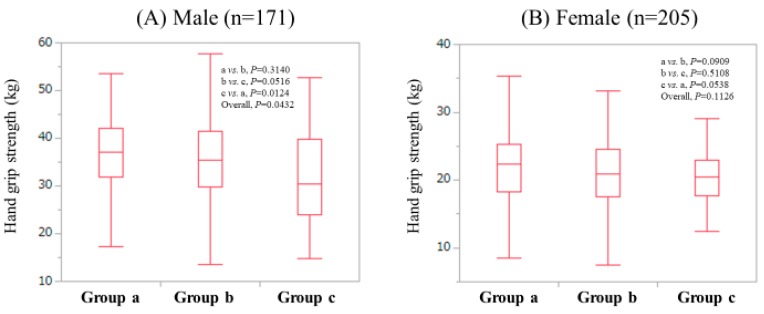
GS value according to BMI in males (**A**) and females (**B**); Group A indicates patients with BMI > 25 kg/m^2^; Group B indicates patients with 20 kg/m^2^ < BMI < 25 kg/m^2^; Group C indicates patients with BMI < 20 kg/m^2^.

Patients were classified into three groups according to BMI: ≥25 kg/m^2^ (Group A); ≥20 kg/m^2^ and <25 kg/m^2^ (Group B); and <20 kg/m^2^ (Group C). In males, the median (IQR) GS in groups of a (*n* = 24), b (*n* = 94) and c (*n* = 53) were: 37.0 kg (31.85 kg, 42.1 kg), 35.5 kg (29.725 kg, 41.525 kg) and 30.35 kg (24.05 kg, 39.85 kg), respectively. The overall difference was noted with significance (*P* = 0.0432: A vs. B, *P* = 0.3140; B vs. C, *P* = 0.0516; A vs. C, *P* = 0.0124). (Figure 3A) In females, the median (IQR) GS in groups of a (*n* = 57), b (*n* = 108) and c (*n* = 40) were: 22.3 kg (18.25 kg, 25.2 kg), 20.9 kg (17.55 kg, 24.475 kg) and 20.4 kg (17.75 kg, 22.975 kg), respectively. Overall differences did not reach significance (*P* = 0.1126: A vs. B, *P* = 0.0909; B vs. C, *P* = 0.5108; A vs. C, *P* = 0.0538). (Figure 3B)

### 3.5. Correlation Between GS and m2bpgi in Male and Female

**Figure 4:**
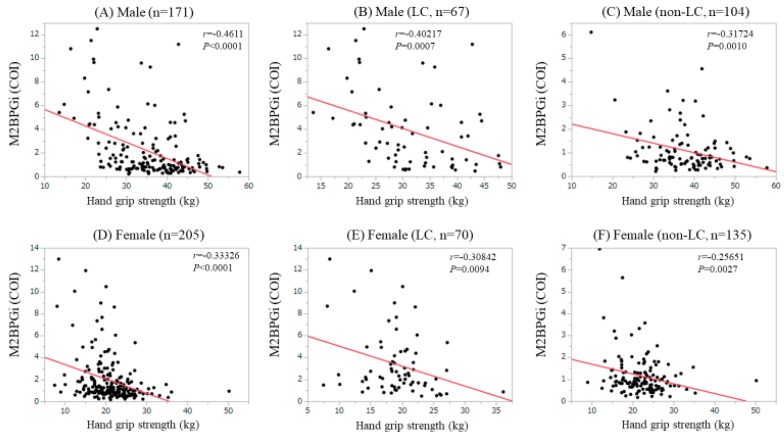
Correlation between GS and Mac-2 binding protein glycosylation isomer (M2BPGi); (**A**) Male cases; (**B**) LC male cases; (**C**) non-LC male cases; (**D**) Female cases; (**E**) LC female case; (**F**) non-LC female cases.

In the males, M2BPGi significantly correlated with GS for all cases (*r* = −0.4611, *P* < 0.0001), LC patients (*r* = −0.40217, *P* = 0.0007) and non-LC patients (*r* = −0.31724, *P* = 0.0010). (Figure 4A–C) Similarly, in the females, M2BPGi significantly correlated with GS for all cases (*r* = −0.33326, *P* < 0.0001), LC patients (*r* = −0.30842, *P* = 0.0094) and non-LC patients (*r* = −0.25651, *P* = 0.0027). (Figure 4D–F).

### 3.6. Correlation Between GS and Fib-4 Index in Male and Female

**Figure 5:**
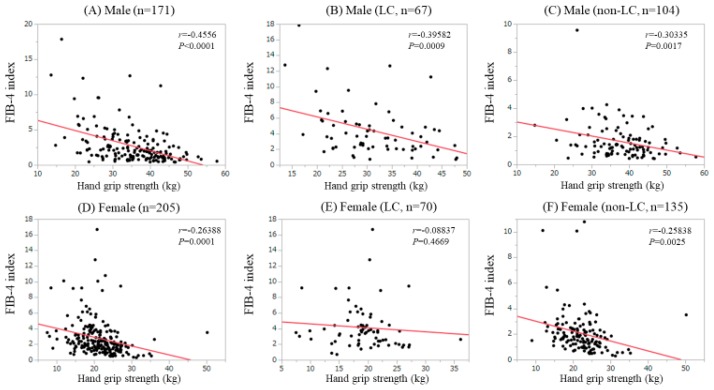
Correlation between GS and the Fibrosis-4 (FIB4) index; (**A**) Male cases; (**B**) LC male cases; (**C**) non-LC male cases; (**D**) Female cases; (**E**) LC female case; (**F**) non-LC female cases.

In males, the FIB-4 index significantly correlated with GS for all cases (*r* = −0.4556, *P* < 0.0001), LC patients (*r* = −0.39582, *P* = 0.0009) and non-LC patients (*r* = −0.30335, *P* = 0.0017). (Figure 5A–C) In females, FIB-4 index significantly correlated with GS for all cases (*r* = −0.26388, *P* = 0.0001) and LC patients (*r* = −0.25838, *P* = 0.0025), while did not for non-LC patients (*r* = −0.08837, *P* = 0.4669). (Figure 5D–F).

### 3.7. Uni- and Multivariate Analyses of Factors Linked to Low GS in Male Patients

In males, the univariate analysis observed ten factors to be significantly associated with low GS (<26 kg): Age (*P* < 0.0001), BMI (*P* = 0.0357), presence of LC (*P* < 0.0001), serum albumin level (*P* < 0.0001), prothrombin time (PT, *P* = 0.0013), platelet count (*P* = 0.0025), M2BPGi (*P* < 0.0001), FIB-4 index (*P* < 0.0001), estimated glomerular filtration rate (*P* = 0.0249) and SMI (*P* = 0.0028). (Table 2) Multivariate analysis for the ten factors showed that M2BPGi (*P* = 0.0003) and SMI (*P* = 0.0007) were significant factors linked to the low GS. (Table 3) Hazard ratios (HRs) and 95% confidence intervals (CIs) for these items were indicated in Table 3. 

### 3.8. Uni- and Multivariate Analyses of Factors Lnked to Low GS in Female Patients

In females, the univariate analysis observed seven factors to be significantly associated with low GS (<18 kg): Age (*P* < 0.0001), serum albumin level (*P* = 0.0209), PT (*P* = 0.0283), platelet count (*P* = 0.0255), M2BPGi (*P* = 0.0017), FIB-4 index (*P* = 0.0038) and SMI (*P* = 0.0036). (Table 4) Multivariate analysis for the seven factors showed that age (*P* < 0.0001) and serum albumin level (*P* = 0.0484) were significant factors linked to the low GS. (Table 5) HRs and 95% CIs for these items were indicated in Table 5. 

## 4. Discussion

Liver and muscle are both metabolically active endocrine organs, and CLDs and sarcopenia, as defined by muscle mass loss and muscle strength decline, may share common pathogenic determinants [32,33]. However, to our knowledge, there have been few studies of factors relevant to muscle strength decline, especially focusing on liver fibrosis markers. There is a high correlation between GS and other muscle strength, and thus GS can be considered as an index of muscle strength throughout the body. Reduced muscle strength, as defined by decreased GS, has been associated with an elevated risk of mortality in many preceding researches [9,34,35,36]. Thus, to elucidate factors associated with GS decline may be clinically meaningful. 

In our data, the median GS values were 35.5 kg in males and 21.1 kg in females. The average GS values in Japanese adults in their 60s are reported to be around 40 kg in males and around 25 kg in females, which are higher than our data [37]. CLD itself may cause these results. In our results, M2BPGi had significant negative correlation with GS irrespective of the LC status or gender, while in the FIB-4 index, similar tendencies were observed except for female LC cases. Our LC patients had significantly low GS compared with non-LC patients, which can explain the significant relationship between GS and liver fibrosis markers. M2BPGi can be a surrogate marker for assessing hepatic stellate cells (HSCs) activation, which are associated with liver fibrosis progression [13]. HSCs are also reported to be involved in extrahepatic disease progression [13]. In CLD patients with low GS, close monitoring for liver fibrosis may be required. While the FIB-4 index includes age [29], as shown in Figure 2A,B, GS decreases with increasing age both in males and females, which can be linked to our current results. 

In our multivariate analysis for male patients, M2BPGi (*P* = 0.0003) and SMI (*P* = 0.0007) were significant factors linked to the low GS. In male CLD patients, M2BPGi rather than the FIB-4 index may affect GS. Sung et al. reported that hepatic encephalopathy, higher M2BPGi, higher age and lower GS were independent predictors of skeletal muscle decline [38]. Our results were consistent with their results. While in our multivariate analysis for female patients, age (*P* < 0.0001) and serum albumin level (*P* = 0.0484) were independent predictors for the low GS. Although the reasons for these discrepancies between genders in the multivariate analyses are unclear, clinicians should be aware that factors influencing on GS can be different between genders. On the other hand, contrary to our expectations, the serum ammonia level was not associated with low GS both in males and females in the univariate analyses. We have previously reported that hyperammonemia in LC patients correlated with a higher serum myostatin level, which strongly suppresses skeletal muscle growth [39]. In our hypothesis, hyperammonemia caused hypermyostatinemia and subsequent muscle strength decline. Further investigations will be needed to confirm the impact of hyperammonemia on GS decline. 

SMI had a significant correlation with GS both in male (*r* = 0.451957, *P* < 0.0001) and female (*r* = 0.427142, *P* < 0.0001) in this study, which suggests the close linkage between muscle strength and muscle mass. In the current guidelines for sarcopenia, muscle function is assessed firstly and in patients with decreased muscle strength, muscle mass is assessed secondly [22,31,40]. 

Thus, clinical implications of decreased muscle mass with preserved muscle strength should be clarified in future studies. On the other hand, in NAFLD patients, GS is shown to correlate with steatosis grade [6,7,8]. Although investigation of the impact of GS on steatosis in NAFLD patients is beyond the scope of our analysis, to confirm these in Japanese NAFLD patients may be essential. 

Several limitations related to the study warrant mention. First, the study was a single-center observational study with a retrospective nature. Second, the study data was derived from a Japanese liver disease population data, and additional investigations on other races are required to further verify and extend the application to other races. Third, GS can vary depending on patients’ daily life activities. Fourth, patients with massive ascites who are potentially involved in the low GS were excluded due to the lack of reliability in the BIA, creating bias. Finally, the interpretation of our results should be done cautiously, since the direction of the association between baseline data and GS remains unclear, due to the cross-sectional nature of our data. Nevertheless, our study results denoted that liver fibrosis markers, especially M2BPGi, can be closely associated with the GS decline. 

## 5. Conclusions

In conclusion, M2BPGi can be a useful marker for the screening of patients with GS decline. 

## Figures and Tables

**Table 1:** Baseline characteristics (*n* = 376).

Variables	Number or
	Median Value (IQR)
Age (years)	64 (52, 71)
Gender, male/female	171/205
HCV/HBV/HCV and HBV/NBNC	208/55/4/109
Body mass index (kg/m^2^)	22.7 (20.7, 25.6)
Hand grip strength (kg, male)	35.5 (29.2, 41.5)
Hand grip strength (kg, female)	21.1 (17.9, 24.4)
Presence of HCC, yes/no	9/367
Presence of LC, yes/no	137/239
Child-Pugh, A/B/C	345/25/6
Total bilirubin (mg/dL)	0.8 (0.6, 1.0)
Serum albumin (g/dL)	4.3 (4.0, 4.5)
Prothrombin time (%)	92.4 (83.825, 101.95)
Platelet count (× 10^4^/mm^3^)	18.0 (12.9, 22.475)
M2BPGi (cutoff index)	1.11 (0.70, 2.34)
FIB4-index	2.069 (1.2515, 3.451)
SMI (kg/m^2^, male)	7.61 (7.07, 8.12)
SMI (kg/m^2^, female)	5.94 (5.505, 6.48)
AST (IU/L)	24 (19, 32)
ALT (IU/L)	49 (14, 30)
HbA1c (NGSP)	5.7 (5.4, 6.0)
eGFR (ml/min/1.73m^2^)	81 (68, 99)
Serum ammonia (μg/dl)	39 (30, 49.5)

HCV: Hepatitis C Virus; HBV: Hepatitis B Virus; NBNC: Non-B and Non-C; HCC: Hepatocellular Carcinoma; LC: Liver Cirrhosis; M2BPGi: Mac-2 Binding Protein Glycosylation Isomer; SMI: Skeletal Muscle Index; NGSP: National Glycohemoglobin Standardization Program; AST: Aspartate Aminotransferase; ALT: Alanine Aminotransferase; eGFR: Estimated Glomerular Filtration Rate; IQR: Interquartile Range.

**Table 2:** Univariate analyses of factors linked to grip strength (GS) < 26 kg in male.

Variables	GS ≥ 26 kg (*n* = 146)	GS < 26 kg (*n* = 25)	*P* Value
Age (years)	58.5 (48, 68)	73 (68, 76.5)	< 0.0001
HBV/HCV/HBV and HCV/NBNC	31/76/2/37	4/14/0/7	0.8614
Body mass index (kg/m^2^)	23.8 (21.8, 26)	21.5 (19.85, 24.75)	0.0357
Presence of LC, yes/no	48/98	19/6	< 0.0001
Total bilirubin (mg/dl)	0.8 (0.6, 1.1)	0.8 (0.6, 1.45)	0.3377
Serum albumin (g/dl)	4.4 (4.1, 4.6)	3.7 (3.05, 4.05)	< 0.0001
Prothrombin time (%)	92.45 (84.2, 99.65)	80.4 (54.8, 93.1)	0.0013
Platelet count (× 10^4^/mm^3^)	18.55 (12.9, 22.225)	13.4 (8.1, 18.0)	0.0025
M2BPGi	0.9 (0.61, 1.7825)	4.93 (2.615, 7.85)	< 0.0001
FIB-4 index	1.73 (0.97, 2.83)	3.89 (2.13, 6.74)	< 0.0001
eGFR (ml/min/1.73m^2^)	84 (71, 100.25)	75 (53, 84)	0.0249
HbA1c (NGSP)	5.6 (5.3, 6.0)	6.0 (5.35, 7.1)	0.0608
Serum ammonia (μg/dl)	43 (33, 54)	49 (33, 74)	0.3551
SMI (kg/m^2^)	7.70 (7.1575, 8.22)	7.12 (6.565, 7.665)	0.0028

HCV: Hepatitis C Virus; HBV: Hepatitis B Virus; NBNC: Non-B and Non-C ; LC: Liver Cirrhosis; M2BPGi: Mac-2 Binding Protein Glycosylation Isomer; SMI: Skeletal Muscle Index; eGFR: Estimated Glomerular Filtration Rate; NGSP: National Glycohemoglobin Standardization Program; SMI: Skeletal Muscle Index.

**Table 3:** Multivariate analyses of factors linked to the low grip strength for male cases.

	Multivariate Analysis		
	Hazard Ratio	95% CI	*P* Value
Age (per one year)	1.015	0.933−1.104	0.7270
BMI (per one kg/m^2^)	0.905	0.688−1.190	0.4754
Presence of LC	2.522	0.448−14.185	0.2940
Serum albumin (per one g/dl)	0.327	0.071−1.502	0.1508
Prothrombin time (per one%)	1.005	0.962−1.050	0.8128
Platelet count (per one × 10^4^/mm^3^)	1.117	0.951−1.311	0.1747
eGFR (per one ml/min/1.73m^2^)	0.990	0.962−1.018	0.4725
M2BPGi (per one COI)	1.740	1.228−2.467	0.0003
FIB−4 index (per one)	1.160	0.797−1.688	0.4344
Skeletal muscle index (per one)	0.124	0.032−0.482	0.0007

BMI: Body Mass Index; LC: Liver Cirrhosis; eGFR: Estimated Glomerular Filtration Rate; M2BPGi: Mac-2 Binding Protein Glycosylation Isomer; COI: Cutoff Index; CI: Confidence Interval.

**Table 4:** Univariate analyses of factors linked to grip strength (GS) < 18 kg in females.

Variables	GS ≥ 18 kg (*n* = 153)	GS < 18 kg (*n* = 52)	*P V*alue
Age (years)	63 (54, 69)	71 (66.25, 75)	<0.0001
HBV/HCV/HBV and HCV/NBNC	17/87/2/47	3/31/0/18	0.5617
Body mass index (kg/m^2^)	22.3 (20.35, 25.45)	21.8 (20.35, 25.25)	0.5834
Presence of LC, yes/no	49/104	21/31	0.3109
Total bilirubin (mg/dl)	0.7 (0.6, 1.0)	0.7 (0.6, 0.9)	0.4856
Serum albumin (g/dl)	4.3 (4.1, 4.5)	4.1 (3.8, 4.4)	0.0209
Prothrombin time (%)	94.5 (86.9, 103.1)	88.6 (82.375, 98.525)	0.0283
Platelet count (×10^4^/mm^3^)	18.9 (13.8, 23.5)	16.25 (10.825, 20.175)	0.0255
M2BPGi (cutoff index)	1.01 (0.685, 1.805)	1.53 (0.8875, 2.845)	0.0017
FIB-4 index	1.983 (1.321, 3.2165)	2.6945 (1.76725, 3.9655)	0.0328
eGFR (ml/min/1.73m^2^)	83 (69, 99)	73 (65.5, 92.5)	0.0759
HbA1c (NGSP)	5.7 (5.4, 6.0)	5.8 (5.4, 6.0)	0.6959
Serum ammonia (μg/dl)	36 (27.75, 42)	33 (21, 45)	0.7235
SMI (kg/m^2^)	6.02 (5.65, 6.53)	5.58 (5.27, 6.33)	0.0036

HCV: Hepatitis C Virus; HBV: Hepatitis B Virus; NBNC: Non-B and Non-C; LC: Liver Cirrhosis; M2BPGi: Mac-2 Binding Protein Glycosylation Isomer; SMI: Skeletal Muscle Index; eGFR: Estimated Glomerular Filtration Rate; NGSP: National Glycohemoglobin Standardization Program; SMI: Skeletal Muscle Index.

**Table 5:** Multivariate analyses of factors linked to the low grip strength for female cases.

	Multivariate Analysis		
	Hazard Ratio	95% CI	*P* Value
Age (per one year)	1.085	1.039–1.133	<0.0001
Serum albumin (per one g/dl)	0.363	0.132–0.998	0.0484
Prothrombin time (per one%)	0.988	0.957–1.020	0.4450
Platelet count (per one × 10^4^/mm^3^)	0.987	0.915–1.064	0.7210
M2BPGi (per one COI)	1.100	0.902–1.343	0.3502
FIB-4 index (per one)	1.129	0.881–1.446	0.3100
Skeletal muscle index (per one)	0.657	0.384–1.125	0.1181

M2BPGi: Mac-2 Binding Protein Glycosylation Isomer; COI: Cutoff Index; CI: Confidence Interval.

## Abbreviations

GS: Grip Strength; CLD: Chronic Liver Disease; NAFLD: Non-alcoholic Fatty Liver Disease; M2BPGi: Mac-2 Binding Protein Glycosylation Isomer; LC: Liver Cirrhosis; SVR: Sustained Virological Response; HCV: Hepatitis C Virus; HCC: Hepatocellular Carcinoma; SMI: Skeletal Muscle Index; BIA: Bioimpedance Analysis; IQR: Interquartile Range; COI: Cutoff Index; HR: Hazard Ratio; CI: Confidence Interval.

## References

[1] Ekerfors U., Sunnerhagen K.S., Westin J., Jakobsson U.E., Marschall H.U., Josefsson A., Simrén M. (2019). Muscle performance and fatigue in compensated chronic liver disease. Scand. J. Gastroenterol..

[2] Nagamatsu A., Kawaguchi T., Hirota K., Koya S., Tomita M., Hashida R., Kita Y., Narao H., Manako Y., Tanaka D. (2019). Slow Walking Speed Overlapped with Low Hand Grip Strength in Chronic Liver Disease Patients with Hepatocellular Carcinoma. Hepatol. Res..

[3] Kulkarni S., Chen H., Josbeno D., Schmotzer A., Hughes C., Humar A., Sood P., Rachakonda V., Dunn M., Tevar A. (2019). Gait Speed and Grip Strength Are Associated With Dropping Out of the Liver Transplant Waiting List. Transplant. Proc..

[4] Nishikawa H., Enomoto H., Yoh K., Iwata Y., Sakai Y., Kishino K., Ikeda N., Takashima T., Aizawa N., Takata R. (2018). Health-Related Quality of Life in Chronic Liver Diseases: A Strong Impact of Hand Grip Strength. J. Clin. Med..

[5] Nishikawa H., Enomoto H., Yoh K., Iwata Y., Sakai Y., Kishino K., Ikeda N., Takashima T., Aizawa N., Takata R. (2018). Effect of Sarcopenia on Sleep Disturbance in Patients with Chronic Liver Diseases. J. Clin. Med..

[6] Kim B.-J., Ahn S.H., Lee S.H., Hong S., Hamrick M.W., Isales C.M., Koh J.-M. (2019). Lower hand grip strength in older adults with non-alcoholic fatty liver disease: A nationwide population-based study. Aging.

[7] Gan D., Wang L., Jia M., Ru Y., Ma Y., Zheng W., Zhao X., Yang F., Wang T., Mu Y. (2019). Low muscle mass and low muscle strength associate with nonalcoholic fatty liver disease. Clin. Nutr..

[8] Lee I., Cho J., Park J., Kang H. (2018). Association of hand-grip strength and non-alcoholic fatty liver disease index in older adults. J. Exerc. Nutr. Biochem..

[9] Leong D.P., Teo K.K., Rangarajan S., Lopez-Jaramillo P., Avezum A., Orlandini A., Seron P., Ahmed S.H., Rosengren A., Kelishadi R. (2015). Prognostic value of grip strength: Findings from the Prospective Urban Rural Epidemiology (PURE) study. Lancet.

[10] Fujiyoshi M., Kuno A., Gotoh M., Fukai M., Yokoo H., Kamachi H., Kamiyama T., Korenaga M., Mizokami M., The Hepatitis Glyco-biomarker Study Group (2015). Clinicopathological characteristics and diagnostic performance of Wisteria floribunda agglutinin positive Mac-2-binding protein as a preoperative serum marker of liver fibrosis in hepatocellular carcinoma. J. Gastroenterol..

[11] Toshima T., Shirabe K., Ikegami T., Yoshizumi T., Kuno A., Togayachi A., Gotoh M., Narimatsu H., Korenaga M., Mizokami M. (2015). A novel serum marker, glycosylated Wisteria floribunda agglutinin-positive Mac-2 binding protein (WFA(+)-M2BP), for assessing liver fibrosis. J. Gastroenterol..

[12] Yamasaki K., Tateyama M., Abiru S., Komori A., Nagaoka S., Saeki A., Hashimoto S., Sasaki R., Bekki S., Kugiyama Y. (2014). Elevated serum levels of Wisteria floribunda agglutinin-positive human Mac-2 binding protein predict the development of hepatocellular carcinoma in hepatitis C patients. Hepatology.

[13] Shirabe K., Bekki Y., Gantumur D., Araki K., Ishii N., Kuno A., Narimatsu H., Mizokami M. (2018). Mac-2 binding protein glycan isomer (M2BPGi) is a new serum biomarker for assessing liver fibrosis: more than a biomarker of liver fibrosis. J. Gastroenterol..

[14] Narimatsu H., Sato T. (2018). Wisteria floribunda agglutinin positive glycobiomarkers: a unique lectin as a serum biomarker probe in various diseases. Expert. Rev. Proteomics..

[15] Toyoda H., Tada T., Yasuda S., Mizuno K., Ito T., Kumada T. (2019). Dynamic Evaluation of Liver Fibrosis to Assess the Risk of Hepatocellular Carcinoma in Patients with Chronic Hepatitis C Who Achieved Sustained Virologic Response. Clin. Infect. Dis..

[16] Seko Y., Moriguchi M., Hara T., Kataoka S., Okuda K., Furuta M., Takemura M., Taketani H., Umemura A., Nishikawa T. (2019). Presence of varices in patients after hepatitis C virus eradication predicts deterioration in the FIB-4 index. Hepatol. Res..

[17] Cheng P.-N., Chiu H.-C., Chiu Y.-C., Chen S.-C., Chen Y. (2018). Comparison of FIB-4 and transient elastography in evaluating liver fibrosis of chronic hepatitis C subjects in community. PLoS ONE.

[18] Tamaki N., Higuchi M., Kurosaki M., Kirino S., Osawa L., Watakabe K., Wang W., Okada M., Shimizu T., Takaura K. (2019). Wisteria floribunda agglutinin-positive mac-2 binding protein as an age-independent fibrosis marker in nonalcoholic fatty liver disease. Sci. Rep..

[19] Ioannou G.N., Beste L.A., Green P.K., Singal A.G., Tapper E.B., Waljee A.K., Sterling R.K., Feld J.J., Kaplan D.E., Taddei T.H. (2019). Increased Risk for Hepatocellular Carcinoma Persists Up to 10 Years After HCV Eradication in Patients with Baseline Cirrhosis or High FIB-4 Scores. Gastroenterology.

[20] Vilar-Gomez E., Chalasani N. (2018). Non-invasive assessment of non-alcoholic fatty liver disease: Clinical prediction rules and blood-based biomarkers. J. Hepatol..

[21] Houot M., Ngo Y., Munteanu M., Marque S., Poynard T. (2016). Systematic review with meta-analysis: direct comparisons of biomarkers for the diagnosis of fibrosis in chronic hepatitis C and B. Aliment. Pharmacol. Ther..

[22] Nishikawa H., Shiraki M., Hiramatsu A., Moriya K., Hino K., Nishiguchi S. (2016). Japan Society of Hepatology guidelines for sarcopenia in liver disease: Recommendation from the working group for creation of sarcopenia assessment criteria. Hepatol. Res..

[23] Fukui H., Saito H., Ueno Y., Uto H., Obara K., Sakaida I., Shibuya A., Seike M., Nagoshi S., Segawa M. (2016). Evidence-based clinical practice guidelines for liver cirrhosis 2015. J. Gastroenterol..

[24] Kudo M., Zheng R.Q., Kim S.R., Okabe Y., Osaki Y., Iijima H., Itani T., Kasugai H., Kanematsu M., Ito K. (2008). Diagnostic Accuracy of Imaging for Liver Cirrhosis Compared to Histologically Proven Liver Cirrhosis. Intervirology.

[25] Zarski J.P., Sturm N., Guechot J., Paris A., Zafrani E.S., Asselah T., Boisson R.C., Bosson J.L., Guyader D., Renversez J.C. (2012). Comparison of nine blood tests and transient elastography for liver fibrosis in chronic hepatitis C: the ANRS HCEP-23 study. J. Hepatol..

[26] Tsochatzis E., Gurusamy K., Ntaoula S., Cholongitas E., Davidson B., Burroughs A., Tsochatzis E., Gurusamy K. (2011). Elastography for the diagnosis of severity of fibrosis in chronic liver disease: A meta-analysis of diagnostic accuracy. J. Hepatol..

[27] Kudo M., Izumi N., Kokudo N., Matsui O., Sakamoto M., Nakashima O., Kojiro M., Makuuchi M., HCC Expert Panel of Japan Society of Hepatology (2011). IN Management of hepatocellular carcinoma in Japan: Consensus-Based Clinical Practice Guidelines proposed by the Japan Society of Hepatology (JSH) 2010 updated version. Dig. Dis..

[28] Hasegawa K., Takata R., Nishikawa H., Enomoto H., Ishii A., Iwata Y., Miyamoto Y., Ishii N., Yuri Y., Nakano C. (2016). Impact of Wisteria floribunda Agglutinin-Positive Mac-2-Binding Protein in Patients with Hepatitis C Virus-Related Compensated Liver Cirrhosis. Int. J. Mol. Sci..

[29] Nishikawa H., Nishijima N., Enomoto H., Sakamoto A., Nasu A., Komekado H., Nishimura T., Kita R., Kimura T., Iijima H. (2017). Comparison of FIB-4 index and aspartate aminotransferase to platelet ratio index on carcinogenesis in chronic hepatitis B treated with entecavir. J. Cancer.

[30] Nishikawa H., Ishii A., Iwata Y., Miyamoto Y., Yuri Y., Takata R., Hasegawa K., Nakano C., Nishimura T., Kazunori Y. (2017). Development of a simple predictive model for decreased skeletal muscle mass in patients with compensated chronic liver disease. Hepatol. Res..

[31] Arai H., Akishita M., Chen L.-K. (2014). Growing research on sarcopenia in Asia. Geriatr. Gerontol. Int..

[32] Sinclair M., Gow P.J., Grossmann M., Angus P.W. (2016). Review article: Sarcopenia in cirrhosis - aetiology, implications and potential therapeutic interventions. Aliment. Pharmacol. Ther..

[33] Lai J.C., Covinsky K.E., McCulloch C.E., Feng S. (2018). The Liver Frailty Index Improves Mortality Prediction of the Subjective Clinician Assessment in Patients With Cirrhosis. Am. J. Gastroenterol..

[34] Ortega F.B., Silventoinen K., Tynelius P., Rasmussen F. (2012). Muscular strength in male adolescents and premature death: cohort study of one million participants. BMJ.

[35] López-Jaramillo P., Cohen D.D., Gomez-Arbelaez D., Bosch J., Dyal L., Yusuf S., Gerstein H.C. (2014). Association of handgrip strength to cardiovascular mortality in pre-diabetic and diabetic patients: A subanalysis of the ORIGIN trial. Int. J. Cardiol..

[36] Sasaki H., Kasagi F., Yamada M., Fujita S. (2007). Grip Strength Predicts Cause-Specific Mortality in Middle-Aged and Elderly Persons. Am. J. Med..

[37] Yoshida D., Suzuki T., Shimada H., Park H., Makizako H., Doi T., Anan Y., Tsutsumimoto K., Uemura K., Ito T. (2014). Using two different algorithms to determine the prevalence of sarcopenia. Geriatr. Gerontol. Int..

[38] Sung J.H., Uojima H., Hidaka H., Tanaka Y., Wada N., Kubota K., Nakazawa T., Shibuya A., Fujikawa T., Yamanoue H. (2019). Risk factors for loss of skeletal muscle mass in patients with cirrhosis. Hepatol. Res..

[39] Nishikawa H., Enomoto H., Ishii A., Iwata Y., Miyamoto Y., Ishii N., Yuri Y., Hasegawa K., Nakano C., Nishimura T. (2017). Elevated serum myostatin level is associated with worse survival in patients with liver cirrhosis. J. Cachex- Sarcopenia Muscle.

[40] Cruz-Jentoft A.J., Bahat G., Bauer J., Boirie Y., Bruyère O., Cederholm T., Cooper C., Landi F., Rolland Y., Sayer A.A. (2019). Sarcopenia: Revised European consensus on definition and diagnosis. Age Ageing.

